# Supplementary material for: Advanced Foot-And-Mouth Disease Vaccine Platform for Stimulation of Simultaneous Cellular and Humoral Immune Responses
Source: Vaccines (Basel). 2020 May 28;8(2):254. doi: 10.3390/vaccines8020254 (PMC7349985; doi:10.3390/vaccines8020254)
Supplement: Supplementary file 1 [file vaccines-08-00254-s001.pdf]

Supplemenatry Materials

A

(A)

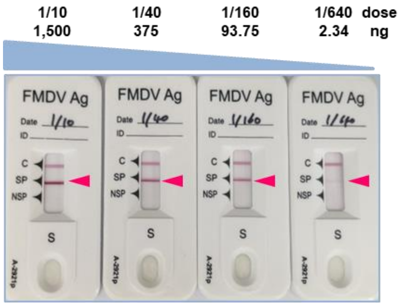

(B)

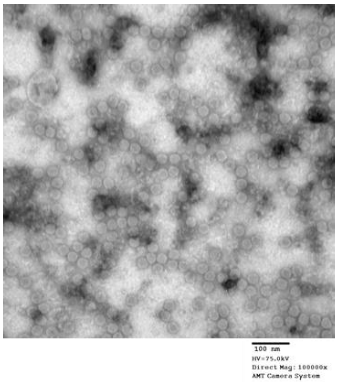

A-3A

(C)

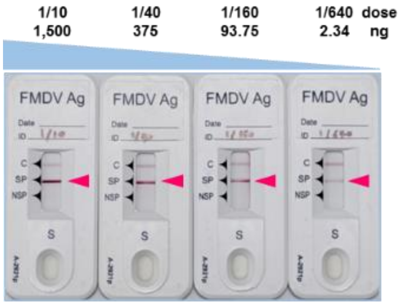

(D)

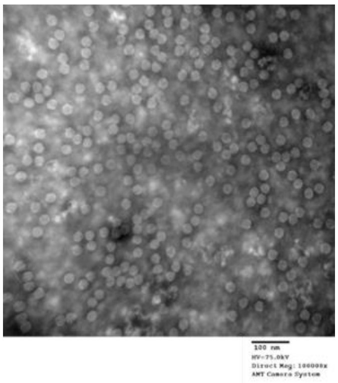

A-HSP70

(E)

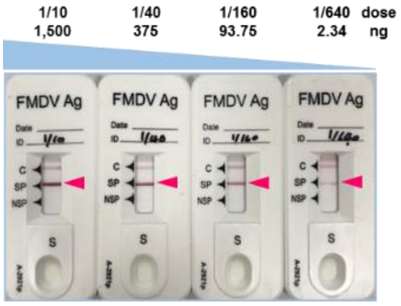

(F)

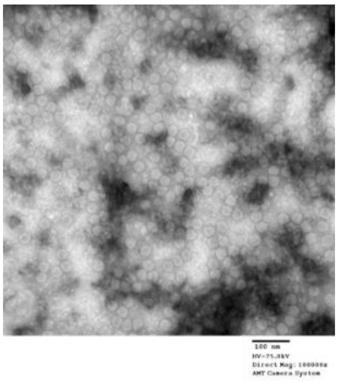

Figure S1. Serially diluted detection of the inactivated FMDV (A-3A, A-HSP70) antigen using a rapid test kit for type A (PBM kit for type A), and electron microscopic examination of inactivated antigen of immunopotent FMD vaccine strains; Structural proteins (SPs) of purified antigen expression of cells infected with immunopotent recombinant FMDV A-3A and A-HSP70 were confirmed by rapid antigen kits (PBM kit) showing band formation for SPs and no band formation for non-structural proteins (NSPs) of FMDV. The virus particle (146S) was characterized by transmission electron microscope (TEM) imaging. (**A–F**) represent (**A**) SP and no NSP band formation of A antigen; (**B**) TEM imaging of A antigen (146S particles); (**C**) SP and no NSP band formation of A-3A antigen; (**D**) TEM imaging of A-3A antigen (146S particles); (**E**) SP and no NSP band formation of A-HSP70 antigen; (**F**) TEM imaging of A-HSP70 antigen (146S particles).

(A)

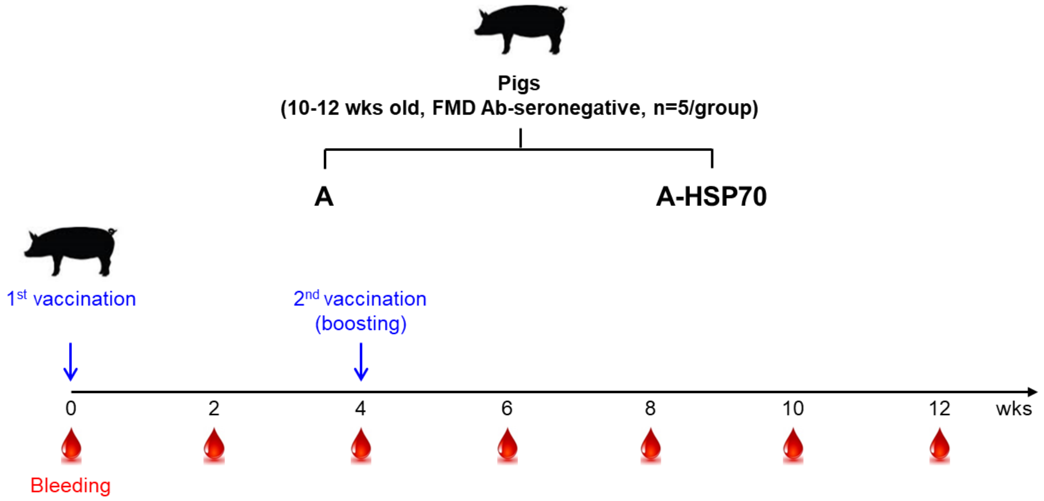

(B)

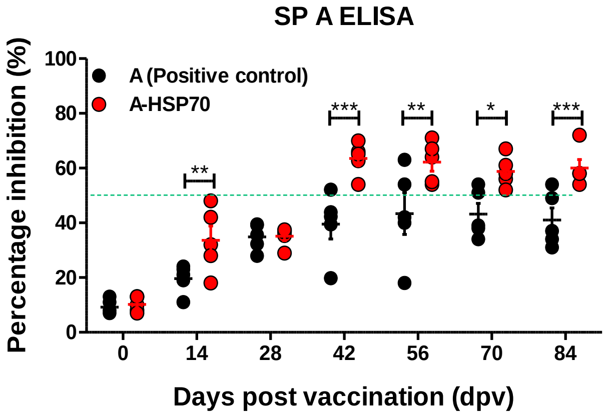

(C)

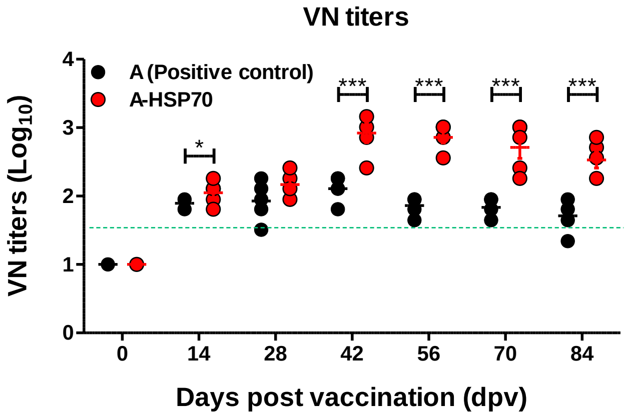

Figure S2. A-HSP70-mediated immune response in pig. Pigs were administered a test vaccine including the A-HSP70 antigen based on the vaccine composition of the positive control group (n = 5/group). The positive control group of pigs received 15 μg (1 dose for pig use) of A antigen, ISA 206 (50%, w/w), 10% $Al(OH)_3$, and 150 μg Quil-A. The vaccination was performed twice at a 28-day interval, and 1 ml of vaccine (1 dose) was injected intramuscularly deep into the animal's necks. Blood samples were collected at 0, 14, 28, 42, 56, 70, and 84 dpv from the pigs for the serological assays. (**A**–**C**) represent (**A**) the strategy for this study; (**B**) antibody titers by SP A ELISA; (**C**) virus-neutralizing antibody titers. The data represent the mean ± SEM of triplicate measurements; statistical analyses

were performed using a two-way ANOVA with a Bonferroni correction; *$p < 0.05$, **$p < 0.01$, ***$p < 0.001$.

Table S1. List of primer sequences for qRT-PCR.

Target	Forward/Reverse	Sequence (5'- 3')	Length (mer)
CD40	CD40 F	GCCAGGACAGAAACTGGTGA	20
	CD40 R	ATTCGCTGGAACTGCAAGGA	20
CD80	CD80 F	TCAGGCATCGTTCAGGTGAC	20
	CD80 R	TGACAGCCAGCACCATTTCA	20
CD86	CD86 F	GTTGTGTGTGGGATGGTGTC	20
	CD86 R	GTTTGTTCACTCGCCTTCCTG	21
MHC Class I	MHC Class I F	GAGTGGACCTGAAGACCCTG	20
	MHC Class I R	CATGCTCTGGAGGGTGTGAG	20
MHC Class II	MHC Class II F	CTCCAGTGATGCTGGGTCAG	20
	MHC Class II R	TGACAGAGTGCCCGTTCTTC	20
CTLA-4	CTLA-4 F	TCTACATGCACTGGCACCTC	20
	CTLA-4 R	CCGTTGCCCATACCCACATA	20
IFNα	IFNα F	CATCTGCTCTCTGGGCTGTG	20
	IFNα R	TGAGGGGATCCAAAGTCCCT	20
IFNβ	IFNβ F	TGCAACCACCACAATTCCAGA	21
	IFNβ R	GGTTTCATTCCAGCCAGTGC	20
IFNγ	IFNγ F	GCCATTCAAAGGAGCATGGAT	21
	IFNγ R	CTGATGGCTTTGCGCTGGAT	20
IL-12p40	IL-12p40 F	CCATATCCAGTGCGGGGATG	20
	IL-12p40 R	AGGCCTTGGTGGATCCTTTG	20
IL-17A	IL-17A F	CTCGTGAAGGCGGGAATCAT	20
	IL-17A R	GGTGTGCTCCGGTTCAAGAT	20
HPRT	HPRT F	GAGGCCATCACATCGTAGCC	20
	HPRT R	TGTAATCCAGCAGGTCAGCA	20

Table S2. Statistical analysis of the data from Figure 2. C57BL/6 mice were administered with the test vaccine, including 1/10, 1/40, 1/160, 1/640 doses of A-3A or A-HSP70 antigen for cattle or pig use, ISA 206 (oil-based emulsion, 50%, w/w), 10% $Al(OH)_3$, and 15 μg Quil-A. A negative control group of mice was injected with the same volume of PBS as the vaccine. The test vaccines were injected intramuscularly into mice that were later challenged with FMDV (100 LD_{50} A/Malay/97) at 7 dpv. The survival rates and body weights were monitored for 7 dpc. (**A**, **B**) represent (**A**) Changes of body weight for A-3A administrated group; (**B**) Changes of body weight for A-HSP70 administrated group. The data are the means ± SEM of triplicate measurements (n = 5/group) and significance was determined using two-way ANOVAs with Bonferroni correction. *$p < 0.05$; **$p < 0.01$; ***$p < 0.001$; ns, not significant.

(A)				
**1 dpc**				
	NC	A-3A (1/10 dose)	A-3A (1/40 dose)	A-3A (1/160 dose)
A-3A (1/10 dose)	ns			
A-3A (1/40 dose)	ns	ns		
A-3A (1/160 dose)	ns	ns	ns	
A-3A	ns	ns	ns	ns

(1/640 dose)				
**2 dpc**				
	NC	A-3A (1/10 dose)	A-3A (1/40 dose)	A-3A (1/160 dose)
A-3A (1/10 dose)	***			
A-3A (1/40 dose)	***	ns		
A-3A (1/160 dose)	**	*	*	
A-3A (1/640 dose)	ns	***	***	ns
**3 dpc**				
	NC	A-3A (1/10 dose)	A-3A (1/40 dose)	A-3A (1/160 dose)
A-3A (1/10 dose)				
A-3A (1/40 dose)		ns		
A-3A (1/160 dose)		ns	ns	
A-3A (1/640 dose)		ns	ns	ns
**4 dpc**				
	NC	A-3A (1/10 dose)	A-3A (1/40 dose)	A-3A (1/160 dose)
A-3A (1/10 dose)				
A-3A (1/40 dose)		ns		
A-3A (1/160 dose)		ns	*	
A-3A (1/640 dose)		***	***	ns
**5 dpc**				
	NC	A-3A (1/10 dose)	A-3A (1/40 dose)	A-3A (1/160 dose)
A-3A (1/10 dose)				
A-3A (1/40 dose)		ns		
A-3A (1/160 dose)		ns	**	
A-3A (1/640 dose)		***	***	**
**6 dpc**				
	NC	A-3A (1/10 dose)	A-3A (1/40 dose)	A-3A (1/160 dose)
A-3A (1/10 dose)				
A-3A		ns		

(1/40 dose)				
A-3A (1/160 dose)		*	**	
A-3A (1/640 dose)		***	***	ns
**7 dpc**				
	NC	A-3A (1/10 dose)	A-3A (1/40 dose)	A-3A (1/160 dose)
A-3A (1/10 dose)				
A-3A (1/40 dose)		ns		
A-3A (1/160 dose)		**	**	
A-3A (1/640 dose)		***	***	ns
(B)				
**1 dpc**				
	NC	A-3A (1/10 dose)	A-3A (1/40 dose)	A-3A (1/160 dose)
A-3A (1/10 dose)	ns			
A-3A (1/40 dose)	ns	ns		
A-3A (1/160 dose)	ns	ns	ns	
A-3A (1/640 dose)	ns	ns	ns	ns
**2 dpc**				
	NC	A-3A (1/10 dose)	A-3A (1/40 dose)	A-3A (1/160 dose)
A-3A (1/10 dose)	***			
A-3A (1/40 dose)	***	ns		
A-3A (1/160 dose)	ns	***	***	
A-3A (1/640 dose)	ns	**	***	ns
**3 dpc**				
	NC	A-3A (1/10 dose)	A-3A (1/40 dose)	A-3A (1/160 dose)
A-3A (1/10 dose)				
A-3A (1/40 dose)		ns		
A-3A (1/160 dose)		**	**	
A-3A (1/640 dose)		**	**	ns
**4 dpc**				

	NC	A-3A (1/10 dose)	A-3A (1/40 dose)	A-3A (1/160 dose)
A-3A (1/10 dose)				
A-3A (1/40 dose)	ns			
A-3A (1/160 dose)	***	**		
A-3A (1/640 dose)	***	**	ns	
**5 dpc**				
	NC	A-3A (1/10 dose)	A-3A (1/40 dose)	A-3A (1/160 dose)
A-3A (1/10 dose)				
A-3A (1/40 dose)	ns			
A-3A (1/160 dose)				
A-3A (1/640 dose)				
**6 dpc**				
	NC	A-3A (1/10 dose)	A-3A (1/40 dose)	A-3A (1/160 dose)
A-3A (1/10 dose)				
A-3A (1/40 dose)	ns			
A-3A (1/160 dose)				
A-3A (1/640 dose)				
**7 dpc**				
	NC	A-3A (1/10 dose)	A-3A (1/40 dose)	A-3A (1/160 dose)
A-3A (1/10 dose)				
A-3A (1/40 dose)	ns			
A-3A (1/160 dose)				
A-3A (1/640 dose)				

Table S3. Statistical analysis of the data from Figure 3. C57BL/6 mice were administrated with oil emulsion free-test vaccines, including A-3A antigen alone, A-HSP70 antigen alone, or a combination of A-3A and A-HSP70 antigens based on their positive control composition. The negative control group received PBS in the same volume as the vaccine. A positive control group received 1.5 μg (1/10 dose for cattle and pig use) of naïve A antigen, without oil emulsion, 10% $Al(OH)_3$, and 15 μg Quil-A without oil emulsion. Briefly, vaccination was performed twice at a 35-day interval; the mice were vaccinated intramuscularly in the thigh muscle. Later, at 84 dpv or 168 dpv, the mice were challenged with FMDV (100 LD_{50} of A/Malay-97, SEA topotype) by intraperitoneal (I.P.) injection. Their survival

rates and body weights were monitored for 7 dpc. (**A**, **B**) represent (**A**) Changes of body weight for challenged group with A/Malay/97 on 84 dpv; (**B**) Changes of body weight for challenged group with A/Malay/97 on 168 dpv. The data are the means ± SEM of triplicate measurements (n = 5/group) and significance was determined using two-way ANOVAs with Bonferroni correction and a one-way ANOVA followed by a Tukey's post-hoc test. *p < 0.05; **p < 0.01; ***p < 0.001; ns, not significant.

(A)				
**1 dpc**				
	NC	A	A-3A	A-HSP70
A	***			
A-3A	ns	***		
A-HSP70	***	ns	**	
A-3A+A-HSP70	ns	***	ns	*
**2 dpc**				
	NC	A	A-3A	A-HSP70
A	*			
A-3A	***	***		
A-HSP70	**	***	***	
A-3A+A-HSP70	***	***	ns	**
**3 dpc**				
	NC	A	A-3A	A-HSP70
A				
A-3A		***		
A-HSP70		***	*	
A-3A+A-HSP70		***	ns	ns
**4 dpc**				
	NC	A	A-3A	A-HSP70
A				
A-3A		***		
A-HSP70		***	ns	
A-3A+A-HSP70		***	ns	ns
**5 dpc**				
	NC	A	A-3A	A-HSP70
A				
A-3A				
A-HSP70			ns	
A-3A+A-HSP70			ns	ns
**6 dpc**				
	NC	A	A-3A	A-HSP70
A				
A-3A				
A-HSP70			ns	
A-3A+A-HSP70			ns	ns
**7 dpc**				
	NC	A	A-3A	A-HSP70
A				
A-3A				
A-HSP70			ns	
A-3A+A-HSP70			ns	ns
(B)				
**1 dpc**				

	NC	A	A-3A	A-HSP70
A	ns			
A-3A	ns	ns		
A-HSP70	ns	ns	ns	
A-3A+A-HSP70	ns	ns	ns	ns
**2 dpc**				
	NC	A	A-3A	A-HSP70
A	***			
A-3A	***	***		
A-HSP70	***	**	ns	
A-3A+A-HSP70	***	*	*	ns
**3 dpc**				
	NC	A	A-3A	A-HSP70
A				
A-3A		***		
A-HSP70		**	ns	
A-3A+A-HSP70		**	ns	ns
**4 dpc**				
	NC	A	A-3A	A-HSP70
A				
A-3A		***		
A-HSP70		***	ns	
A-3A+A-HSP70		**	*	ns
**5 dpc**				
	NC	A	A-3A	A-HSP70
A				
A-3A				
A-HSP70			ns	
A-3A+A-HSP70			ns	ns
**6 dpc**				
	NC	A	A-3A	A-HSP70
A				
A-3A				
A-HSP70			ns	
A-3A+A-HSP70			*	ns
**7 dpc**				
	NC	A	A-3A	A-HSP70
A				
A-3A				
A-HSP70			ns	
A-3A+A-HSP70			*	ns
